# Persistent Neopharynx Pseudomonas Infection After a Sealed Anastomotic Leak Post Total Laryngectomy

**DOI:** 10.7759/cureus.29802

**Published:** 2022-10-01

**Authors:** Marina Mat Baki, Mohd Syafeeq Mohd Ridzam, Norazila Abdul Rahim

**Affiliations:** 1 Department of Otorhinolaryngology - Head and Neck Surgery, Faculty of Medicine, Universiti Kebangsaan Malaysia Medical Centre, Kuala Lumpur, MYS; 2 Department of Otorhinolaryngology - Head and Neck Surgery, Faculty of Medicine, Universiti Teknologi MARA (UiTM) Sungai Buloh Campus, Selangor, MYS

**Keywords:** pseudomonas, neopharynx, pharyngocutaneous fistula, salvage total laryngectomy, anastomotic leak

## Abstract

Total laryngectomy (TL) is the treatment of choice for advanced glottic cancer. Post-operative complications can be debilitating for patients, family members and healthcare workers. Complications following TL have been reported in many studies, with pharyngocutaneous fistula and wound infection being the most common. Identifying the risk factors that may give rise to these complications is vital to minimise post-operative morbidity. We present the case of a 62-year-old male who underwent salvage TL following radiation therapy for recurrent glottic carcinoma. The patient developed diffuse submental swelling upon the commencement of oral feeding. A flexible nasopharyngolaryngoscopy revealed a sloughy area at the neopharynx, with the finding of a sealed anastomotic leak on a repeat barium swallow study. We report persistent *Pseudomonas aeruginosa* infection following salvage TL, after a sealed anastomotic leak.

## Introduction

Head and neck cancers account for about 5.3% of all cancers reported worldwide [1]. Laryngeal cancer ranks second after oral and lip cancers [1]. Cigarette smoking remains the major risk factor in developing larynx cancer, followed by alcohol ingestion, exposure to asbestos or sulphuric acid, and, to a lesser extent, human papillomavirus infection [1,2].

Total laryngectomy (TL) is the gold standard for advanced glottic cancer and salvage surgery [3]. Primary TL is often performed with bilateral neck dissection, with or without adjuvant radiotherapy. It has a high survival rate in patients with advanced glottic carcinoma [4]. Patients who do not respond to oncological treatment are indicated for salvage TL, which causes higher complication rates compared with primary TL [5].

Common complications following TL are pharyngocutaneous fistula (PCF), wound infection, haemorrhage or haematoma, tracheostoma stenosis, chyle leaks, free flap failure and even death [5]. PCF is more common in salvage TL due to sequelae of infections in the anastomotic area. We report the case of a patient who underwent salvage TL complicated with persistent *Pseudomonas aeruginosa* infection of the neopharynx following a sealed anastomotic leak.

## Case presentation

A male patient in his sixties, a chronic smoker with a background history of hypertension, chronic renal failure, ischaemic heart disease and depressive disorder, was diagnosed with glottic carcinoma (T1bN0M0) in March 2019 and underwent radiotherapy with a dose of 66 grays in 33 fractions. Unfortunately, he developed recurrent disease about a year later after serial surveillance imaging showed clearance of the disease. He underwent salvage TL, total thyroidectomy with preservation of the left parathyroid gland, right modified radical neck dissection and left selective neck dissection (levels II, III and IV). Pre-operatively, blood investigation showed a haemoglobin level of 11.8 g/dL, with albumin of 2.9 g/dL. The neopharynx was reconstructed using Vicryl 3/0 in two layers of a continuous suture. Intraoperative assessment with 50 cc diluted methylene blue dye showed no evidence of an anastomotic leak. The histopathological examination (HPE) reported a well-differentiated keratinising squamous cell carcinoma of the larynx (pT4aN0) with no margin involvement.

**Figure 1:**
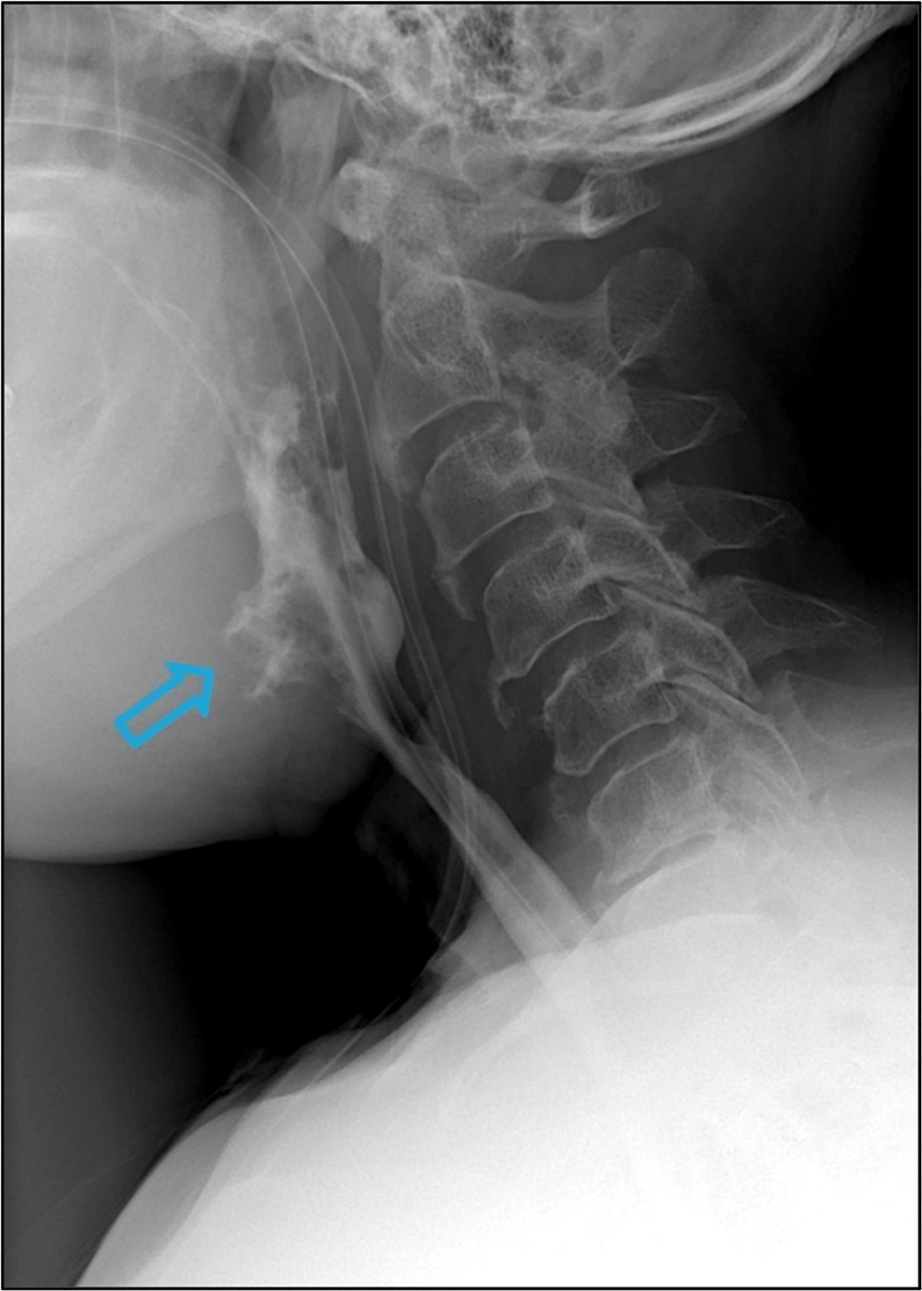
The blue arrow in the barium swallow study demonstrates outpouching with an irregular mucosal outline at the anterior aspect of the neopharynx at the C3/C4 level with no extraluminal contrast extravasation.

**Figure 2:**
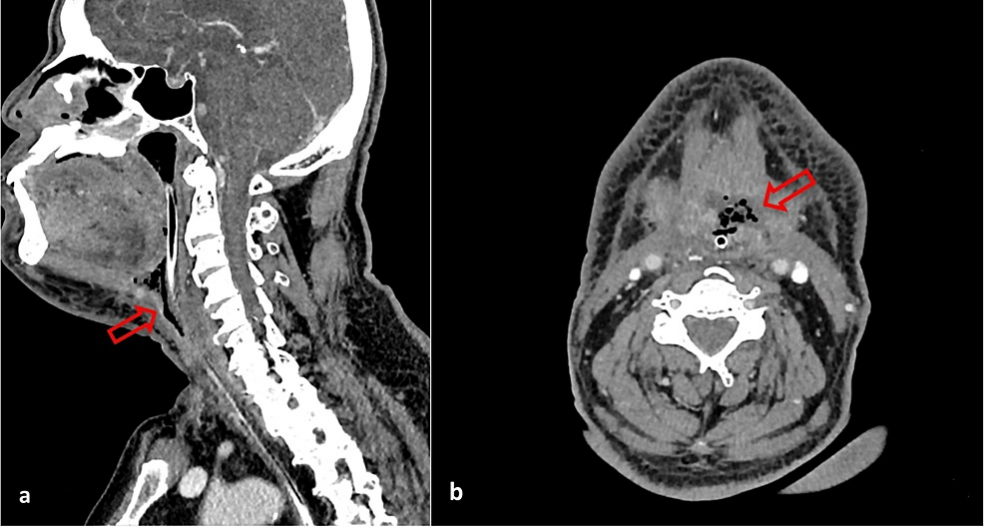
The red arrows in the (a) sagittal and (b) axial CT scan images show multiloculated, rim-enhancing collection anterior to the neopharynx, extending to the submental region at the C3/C4 level and measuring approximately 1.5 cm × 3.3 cm × 1.5 cm.

The nasogastric tube was removed on day 10 post-operatively after a barium swallow revealed no anastomotic leak. One month after TL, the patient developed submental swelling extending to the submandibular region, which started to develop after a week of oral feeding. Clinically, the submental region was diffusely swollen, tender on deep palpation and firm in consistency with inflamed skin, but no discharge or wound breakdown was seen. A flexible nasopharyngolaryngoscopy was performed in the clinic, which revealed a sloughy area at the neopharynx, giving suspicion of an anastomotic leak. Ultrasound of the neck revealed a submental collection with echogenic dermis within it. A repeated barium swallow study showed features that may have represented a contained leak at the anastomotic site, with no demonstrable extraluminal contrast extravasation seen (Figure 1). A contrast-enhanced CT scan of the neck revealed multi-local collections at the submental region anterior to the neopharynx at the C3/C4 level with a sealed-off collection (Figure 2). Pus culture and sensitivity obtained from the sloughy area were reported as *P. aeruginosa* infection.

**Figure 3:**
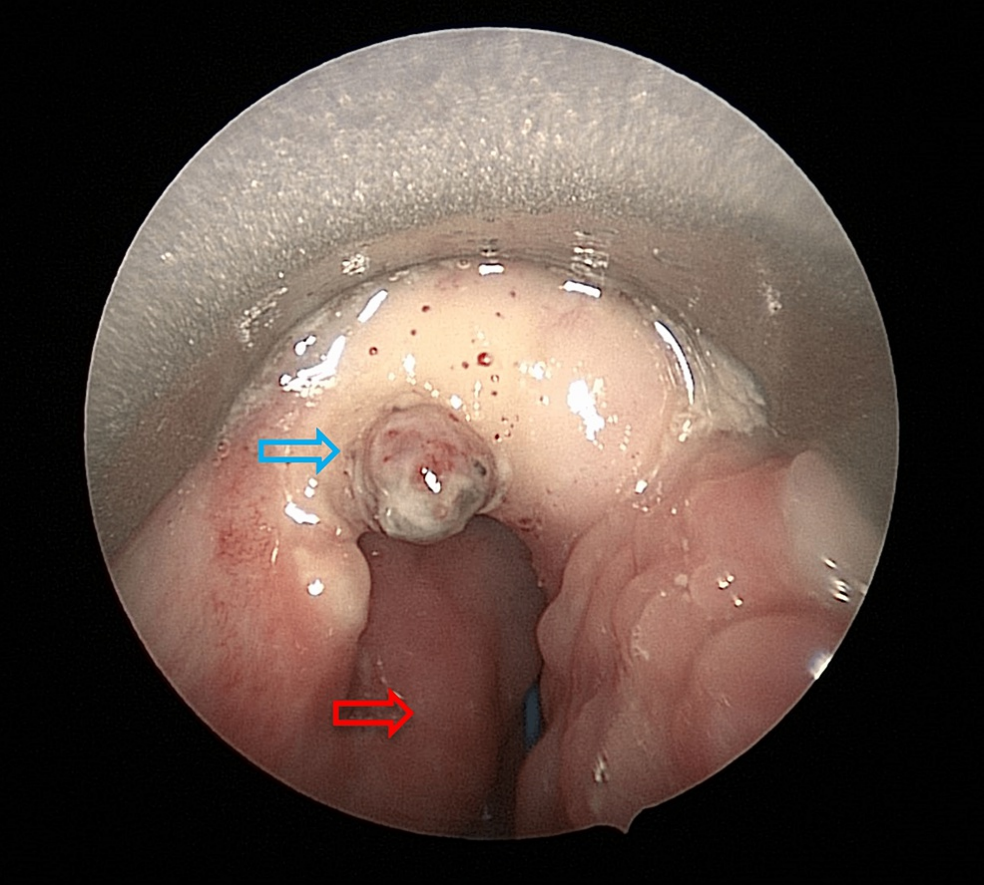
The blue arrow shows a sloughy area at the left lateral part of the base of the tongue. The red arrow shows the posterior pharyngeal wall.

**Figure 4:**
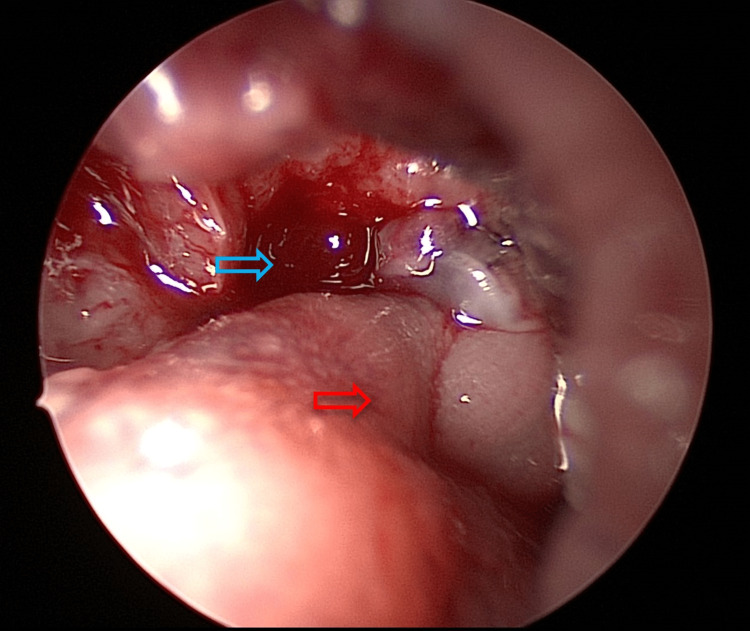
The blue arrow shows the left lateral part of the base of the tongue after debridement of the slough and the tissue biopsy. The red arrow shows the posterior pharyngeal wall.

The patient was then treated with a one-week course of intravenous Ceftazidime and another week of oral Ciprofloxacin. Subsequently, the infection settled with evidence of resolved collection at the submental region on a repeated neck ultrasound and barium swallow study. He was on nasogastric tube feeding while on treatment. One month later, the patient presented with unprovoked peroral bleeding. CT angiography of the carotid showed no evidence of active bleeding. The patient was subjected to direct laryngoscopy and examination under general anaesthesia about three months after TL. Intraoperatively, an area of the slough was seen at the left lateral part of the base of the tongue with no active bleeding point identified (Figure 3). Debridement of the sloughy area was performed, and tissue samples were obtained from the region where the tissue HPE was reported to be negative for malignancy (Figure 4). The tissue culture revealed the same organism as reported previously, *P. aeruginosa*. The patient completed another course of antibiotic treatment, and the infection subsequently resolved.

## Discussion

Following TL, complications range from about 20% to 40%, with the commonest being PCF (3%-65%), followed by wound infections (9%-14%) [6,7]. In post-primary TL, the infection rate ranges between 5% and 10%, while in salvage TL, the infection rate is higher at about 40% to 61% [5,7]. A study by Scotton et al. showed that the most common organisms in post-TL infection are methicillin-resistant *Staphylococcus aureus*, followed by *P. aeruginosa* [7]. Conversely, a study by Le Flem et al. found that *P. aeruginosa* is the most common organism associated with post-TL infection, followed by *S. aureus* [8].

In this case, the patient developed submental swelling extending to the submandibular region one month after TL. The symptom developed one week following the commencement of oral feeding, which gave high suspicion of an anastomotic leak of the neopharynx despite a normal barium swallow study. Both repeat barium swallow study and CT scan demonstrated a contained anastomotic leak with no extraluminal contrast extravasation. Thick secretion aspirated from the anastomotic area at the base of the tongue for pus culture and sensitivity revealed *P. aeruginosa* infection.

Several factors can predispose patients to PCF post-operatively. A previous history of radiation or chemoradiation therapy and multiple comorbidities are the main reported risk factors [7]. Other risk factors include low albumin (less than 3.5 g/dL) and low haemoglobin (less than 12.5 g/dL) levels, a pre-existing tracheostomy tube and concurrent bilateral neck dissection [9]. In this study, a previous history of an irradiated neck, a haemoglobin level of 11.8 g/dL, a low albumin level of 2.9 g/dL in a few readings pre- and post-operatively and bilateral neck dissection increased this patient’s susceptibility to developing a post-TL anastomotic leak.

A poor surgical technique during the reconstruction of the neopharynx may also lead to an anastomotic leak and, eventually, PCF. Intraoperative assessment using a diluted methylene blue dye solution is recommended to assess mucosa integrity after neopharyngeal repair. Daniel et al. reported that 12.9% of patients who had intraoperative leaks during dye tests with immediate neopharyngeal repair had no subsequent incidence of leakage post-operatively [10]. Oral feeding is normally withheld for about a week or longer until no leakage is confirmed with a barium swallow study. Meulemans et al. advised their post-salvage TL patient for a gradual progression of oral intake, starting with clear fluid on the first day after a satisfactory barium swallow followed by liquid and semi-liquid food for the next three weeks. From their study, 15% of salvage TL patients had a delayed PCF after starting peroral feeding even after a satisfactory barium swallow [11]. On the contrary, Le Flem et al. proved that early oral hydration with a sip of water three times a day with prior povidone mouthwash on day 2 after surgery had a lower incidence of developing PCF compared with another group of patients who only commenced oral hydration two weeks post-operatively [8]. In this case, a barium swallow study, which was performed on day 10 after TL, reported no anastomotic leak; therefore, the next day after a trial of safe oral feeding, the nasogastric tube was removed. Despite being reported as a normal study, the patient’s symptoms started to develop after one week of oral feeding.

Although PCF is a common complication after TL, other possibilities, such as residual disease, must be excluded. In this case, the cause of the sealed anastomotic leak with persistent *P. aeruginosa* infection was multifactorial, which may have included the previously irradiated neck, multiple comorbidities, concurrent bilateral neck dissection or inadequate optimisation of blood parameters pre- and post-operatively.

## Conclusions

We conclude that concealed anastomotic leak in a post-salvage TL may cause delayed infection even after a satisfactory barium swallow study. We advised a gradual progression of oral intake with a serial follow-up to prevent such complications, particularly in salvage TL. Nevertheless, residual tumours must be excluded in such cases. Early examination under anaesthesia for debridement, biopsy, and culture and sensitivity study is pertinent to expedite diagnosis and the healing process.
